# Anti-IL-6 therapies in central nervous system inflammatory demyelinating diseases

**DOI:** 10.3389/fimmu.2022.966766

**Published:** 2022-10-27

**Authors:** Li Jiao, Shougang Guo

**Affiliations:** ^1^ Department of Neurology, Shandong Provincial Hospital Affiliated to Shandong First Medical University, Jinan, China; ^2^ Department of Neurology, Shandong Provincial Hospital Affiliated to Shandong University, Jinan, China

**Keywords:** IL-6, central nervous system inflammatory demyelinating diseases, tocilizumab, satralizumab, monoclonal antibodies

## Abstract

Current treatments for central nervous system (CNS) inflammatory demyelinating diseases (IDDs) include corticosteroids, plasma exchange, intravenous immunoglobulin, and immunosuppressant drugs. However, some patients do not respond well to traditional therapies. In recent years, novel drugs, such as monoclonal antibodies, targeting the complement component C5, CD19 on B cells, and the interleukin-6 (IL-6) receptor, have been used for the treatment of patients with refractory CNS IDDs. Among these, tocilizumab and satralizumab, humanized monoclonal antibodies against the IL-6 receptor, have shown beneficial effects in the treatment of this group of diseases. In this review, we summarize current research progress and prospects relating to anti-IL-6 therapies in CNS IDDs.

## Introduction

Central nervous system (CNS) inflammatory demyelinating diseases (IDDs) comprise a heterogeneous group of disorders that mainly includes clinically isolated syndrome, multiple sclerosis (MS), neuromyelitis spectrum disorders (NMOSDs), myelin-oligodendrocyte glycoprotein antibody-associated disease (MOGAD), and acute disseminated encephalomyelitis. These disorders are characterized by myelin loss and axonal damage associated with inflammatory lesions (1–5).

Current treatments for CNS IDDs are primarily aimed at relieving acute attacks and preventing relapse. High-dose corticosteroids, plasma exchange, and intravenous immunoglobulin are frequently employed for the treatment of acute attacks in CNS IDDs (6–14), whereas immunosuppressant drugs, such as oral corticosteroids, azathioprine (AZA), and mycophenolate mofetil, are commonly used for relapse prevention in NMOSDs and MOGAD (15–19). Disease-modifying drugs, such as IFN-β, dimethyl fumarate, and glatiramer acetate, are recommended as mainstream treatments for MS (20, 21). However, some patients do not respond well to these traditional therapies. Over recent years, novel drugs, such as monoclonal antibodies targeting the complement C5 protein, CD19 on B cells, and the interleukin-6 (IL-6) receptor have been used for the treatment of patients with refractory CNS IDDs (22–24).

IL-6 is a cytokine that plays a key role in host defenses, and dysregulated IL-6 signaling is implicated in various autoimmune and inflammatory diseases (25–27). IL-6 signals are transmitted *via* two routes, namely, the classical and trans-signaling pathways. In the classical pathway, IL-6 binds to the membrane-bound IL-6 receptor, forming a complex that recruits glycoprotein 130. In the trans-signaling pathway, IL-6 binds to the soluble form of the IL-6 receptor, and then to membrane-anchored glycoprotein 130 (28, 29). During the adaptive immune response, IL-6 exerts its effects *via* stimulating B-lymphocyte differentiation, promoting antibody production, modulating blood–brain barrier permeability, and enhancing T-lymphocyte activation (30–32). Studies demonstrate that serum and cerebrospinal fluid (CSF) IL-6 levels are significantly increased in patients with NMOSD, whereas IL-6 inhibition is shown to mitigate disease progression (33–38). Several studies have also identified a positive association between serum IL-6 receptor levels and the risk for MS (39–41). Accordingly, IL-6 receptor blockade may represent a novel therapeutic approach for the prevention of relapse in CNS IDDs. Tocilizumab and satralizumab, humanized monoclonal antibodies against the IL-6 receptor, have recently been shown to elicit beneficial clinical effects in the treatment of CNS IDDs (42–46). In this review, we summarize current studies regarding the effects of tocilizumab and satralizumab in the treatment of these disorders.

## Tocilizumab

### Tocilizumab in NMOSD

In 2013, Araki et al. reported the case of a 36-year-old woman who experienced an improvement in her disability burden and neuropathic pain 6 months after tocilizumab therapy (44). The same group described another case series involving seven patients with NMOSD who received monthly injections of tocilizumab (8 mg/kg) for 12 months. The authors reported a significant reduction in the annualized relapse rate (ARR), the Expanded Disability Status Scale (EDSS) score, neuropathic pain, and general fatigue among the patients. The anti-AQP4 antibody titers were also decreased 6 and 12 months after tocilizumab treatment (45).

In a German retrospective observational study in which the patients were followed up for a mean of 30.9 months, eight patients who received tocilizumab displayed a marked decrease in ARR and EDSS scores. Their AQP4 antibody titers and pain levels were also significantly reduced during tocilizumab treatment (46). Subsequently, data from a clinical study confirmed the long-term efficacy and safety of tocilizumab. Nineteen patients were given tocilizumab monthly, and the number of relapses decreased in all cases. Among 15 patients who received tocilizumab for more than 1 year, the EDSS score, ARR, neuropathic pain, and general fatigue were all significantly improved (47). Similar results were reported for 12 NMOSD patients who received at least 6 months of subcutaneous tocilizumab (48). In the same study, both median and annualized relapse rates were significantly decreased (from 2 to 0). The efficacy of subcutaneous tocilizumab appears to render it an alternative to infusion for patients with NMOSD. In Spain, an observational, retrospective study analyzed the effectiveness and safety of tocilizumab in five NMOSD patients who failed to respond to other immunosuppressant drugs. The authors reported that the mean ARR was reduced by 88.9% during the first year of treatment (from 1.8 ± 1.3 to 0.2 ± 0.4, *P*<.05) (49).

The TANGO trial (NCT03350633) was a randomized, open-label phase II trial that included 118 patients who were followed up for 90 weeks at six hospitals in China (50). The patients were randomly given tocilizumab (8 mg/kg every 4 weeks) or AZA (2–3 mg/kg per day). The median time to first relapse was longer in the patients treated with tocilizumab than in those administered AZA (78.9 weeks *vs*. 56.7 weeks; *P*=.0026). In the per-protocol analysis, 89% of the patients treated with tocilizumab were relapse-free after 60 weeks of treatment compared with 56% for AZA treatment (*P*<.0001).

A meta-analysis of five clinical trials that included 89 patients reported that the ARR was significantly reduced in patients treated with tocilizumab. Moreover, a significant correlation was found between the proportion of relapse-free patients and tocilizumab treatment (51). Another meta-analysis involving 775 patients from seven randomized controlled trials found that patients treated with tocilizumab or satralizumab exhibited significantly lower EDSS scores compared with patients treated with other monoclonal antibodies (52). Meanwhile, in a meta-analysis comprising a total of 202 patients with NMOSD from nine studies, Kharel et al. found that 76% of the patients treated with tocilizumab were relapse-free and the ARR was significant reduced (mean difference: −2.6) at follow-up (ranging from 12 to 31.8 months) (53).

Recently, Du et al. undertook a retrospective study on the effects of tocilizumab in 19 NMOSD patients with moderate-to-severe myelitis. The authors found that disease disability scores were significantly improved in patients treated with tocilizumab when compared with those in patients treated with steroids at 3 months. In addition, compared with steroids, tocilizumab treatment led to a significantly lower ARR and risk of relapse (54). A longitudinal study investigated retinal damage in 50 patients with NMOSD who received disease-modifying drugs and reported that, compared with AZA, tocilizumab and rituximab could delay macular ganglion cell complex thinning in the eyes of patients without a history of optic neuritis (55).

### Tocilizumab In MOGAD

In 2019, Novi et al. reported that a 20-year-old patient with MOGAD experienced clinical deterioration despite receiving rituximab treatment, following which he received tocilizumab infusion every 4 weeks. At the 24-month follow-up, the patient was relapse-free, and a spinal MRI showed a reduction in cervical and thoracic lesions (56). Subsequently, Hayward-Koennecke et al. described the case of a 59-year-old man with recurrent optic neuritis who had received high-dose steroids, plasmapheresis, rituximab, natalizumab, and cyclophosphamide due to disease deterioration. Following further relapse, the patient tested positive for anti-MOG antibodies. Tocilizumab was initiated for 12 months and then tapered to an application every 6 weeks. No further relapses occurred (57).

Masuccio et al. reported a patient with MOGAD who experienced severe acute respiratory syndrome coronavirus (SARS-CoV-2) infection during tocilizumab therapy. Given the high risk of relapse, the patient continued taking tocilizumab, and the symptoms stabilized. The patient also retained walking ability (58).

A retrospective study analyzed seven patients with inflammatory CNS disorder (four with NMOSD and positive for anti-AQP4 antibodies and three with MOGAD) who were treated with tocilizumab. The median follow-up period was 23 months (4–50 months). All the patients were relapse-free throughout tocilizumab treatment (59). Similarly, a single-center report involving 10 patients with relapsing MOGAD who received intravenous or subcutaneous tocilizumab found that none of the patients had clinical or radiographic recurrence over an average treatment duration of 28.6 months (60).

Recently, a retrospective study described 57 patients, including 14 with MOGAD, 36 with AQP4-IgG-seropositive NMOSD, and seven with AQP4-IgG-negative NMOSD who switched to tocilizumab from other immunotherapies. The authors reported that 60% of all the patients were relapse-free (79% for patients with MOGAD) during tocilizumab treatment. For MOGAD, the median ARR decreased from 1.75 to 0, and the inflammatory activity on MRI also decreased significantly under tocilizumab treatment. Eleven of the patients with MOGAD who received tocilizumab for more than 12 months had reduced ARR, and 73% of these were relapse-free (61).

### Tocilizumab in MS

To date, relatively few studies have focused on tocilizumab therapy in MS, and the efficacy of tocilizumab as a treatment for this disease remains unclear. In 2014, Sato et al. reported the case of a 53-year-old Japanese woman with MS and rheumatoid arthritis (RA) who received tocilizumab and was relapse-free for more than 5 years (62). Moreover, in 2020, the neurological condition of a Japanese boy who was diagnosed with MS with a tumefactive lesion in the cervical spine deteriorated when his oral prednisolone dose was tapered off. After tocilizumab treatment, the prednisolone dose was reduced without symptom exacerbation, and the EDSS score effectively improved from 8.5 to 5.0 points (63).

## Satralizumab

### Satralizumab in NMOSD

Satralizumab is another IL-6 receptor-targeting monoclonal antibodies. It has better pharmacokinetic properties and a longer half-life than tocilizumab, resulting from modifications in the constant and variable regions of the antibody (64–66).

SAkuraStar (NCT02028884) and SAkuraSky (NCT02073279) are two randomized, double-blind, placebo-controlled phase III studies that compared the efficacy and safety of satralizumab as, respectively, an add-on treatment to other immunosuppressants and as monotherapy in patients with NMOSD (67, 68). In the two trials, 120 mg subcutaneous satralizumab or placebo were administered at weeks 0, 2, and 4 and then every 4 weeks thereafter. The primary endpoint was time to the first protocol-defined relapse. Secondary endpoints were changes in the Functional Assessment of Chronic Illness Therapy–Fatigue score and visual analog scale pain score.

In the SAkuraStar trial, the rate of relapse (8/41, 20%) was lower in the satralizumab group than in the group given the placebo (18/42, 43%). Additionally, 89% and 78% of patients treated with satralizumab were still relapse-free after 48 and 96 weeks, respectively. Among 55 AQP4-IgG-seropositive patients, the relapse rate was 11% in the satralizumab group *versus* 43% in the placebo group. No significant difference between satralizumab and placebo was observed in the AQP4-IgG seronegative subgroup (67). In the SAkuraSky trial, satralizumab elicited a 55% reduction in the relapse risk compared with the placebo. At 48 and 96 weeks, 76% and 72% of patients were relapse-free in the satralizumab group compared with 62% and 51%, respectively, in the placebo group. For the AQP4-IgG-seropositive subgroup, relapse occurred in 22% of patients who received satralizumab *versus* 57% for the placebo. No significant difference was observed between satralizumab and placebo in the AQP4-IgG-negative subgroup (68). Similarly, no significant differences in neuropathic pain or fatigue were detected in either study.

Based on the positive results of these two phase III clinical trials, on June 1, 2020, Health Canada approved satralizumab for use in the treatment of AQP4-IgG-seropositive NMOSD in adults and children aged ≥2 years. Subsequently, satralizumab was also approved in Japan on June 29, 2020, and in Switzerland on July 13, 2020 (69).

Recently, a Japanese study reported a patient with AQP4-IgG-seropositive NMOSD whose painful tonic seizures disappeared after 6 months of satralizumab treatment (70).

## Adverse events

Overall, anti-IL-6 therapy is well-tolerated by patients with NMOSD. Infections, anemia, leukocytopenia, and hypercholesterolemia are the main adverse events associated with tocilizumab treatment in NMOSD (44); however, most are mild, and serious adverse events are rarely reported. Although a meta-analysis found that two patients with NMOSD died during tocilizumab therapy, neither death was related to tocilizumab treatment (50). Most adverse events related to satralizumab therapy are mild to moderate. The most commonly reported adverse events are upper respiratory tract infections, urinary tract infections, nasopharyngitis, and headaches. No deaths occurred during satralizumab treatment (67, 68, 71, 72).

## Perspectives and challenges

### Are IL-6 receptor antagonists also suitable as treatments for MOGAD and MS, in addition to NMOSD?

**Figure 1:**
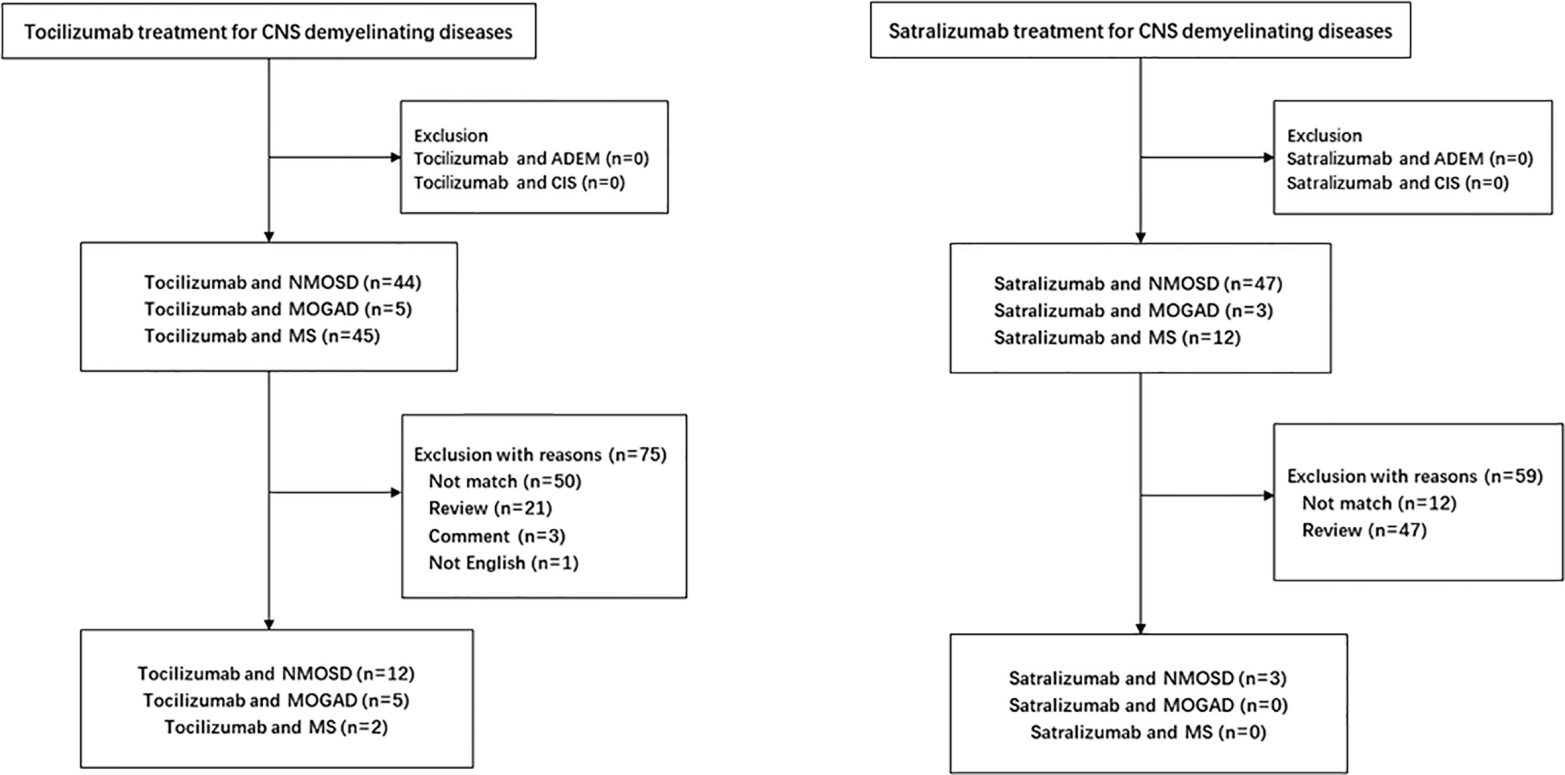
Flowchart enrolled in the study.

Growing evidence supports that IL-6 plays a key role in the pathophysiology of NMOSD. *In vitro*, dysregulated IL-6 activity leads to reduced blood–brain barrier function, increased leukocyte migration, enhanced chemokine production, and activation of AQP4 antibody secretion (32, 63, 73). *In vivo*, serum and CSF levels of IL-6 were found to be elevated in relapsing patients with NMOSD (33, 36, 66). Several studies report the efficacy and safety of IL-6 receptor inhibitor therapy in the treatment of NMOSD (**Figure 1**, **Table 1**).

**Table 1:** Summary of the main clinical trials on tocilizumab and satralizumab in central nervous system inflammatory demyelinating diseases.

Reference	Year	Disease	Patients (*n*)	Treatment	Duration	Study design and outcomes
Araki et al.	2013	AQP4 Ab-positive NMOSD	1	Intravenous TCZ at 8 mg/kg was given monthly	6 months	Case report Serum anti-AQP4 autoantibody levels were rapidly reduced Neuropathic pain and disability scores gradually improved TCZ was well tolerated
Araki et al.	2014	AQP4 Ab-positive NMOSD	7	Intravenous TCZ at 8 mg/kg was given monthly	12 months	Prospective study The ARR decreased from 2.9 ± 1.1 to 0.4 ± 0.8 after 12 months (*P*<0.005) The EDSS score, neuropathic pain, and general fatigue also exhibited a marked declined TCZ was well tolerated
Ringelstein et al.	2015	AQP4 Ab-positive NMOSD	8	Intravenous TCZ at 6 or 8 mg/kg was given monthly	30.9 months	Retrospective study The median ARR decreased significantly (from 4.0 to 0.4; *P*=0.008) as did the median EDSS score (from 7.3 to 5.5; *P*=0.03) Active magnetic resonance imaging lesions were seen in 6 of 8 patients at tocilizumab initiation and in 1 of 8 patients at the last magnetic resonance imaging The AQP4 Ab titers and pain levels dropped significantly during TCZ treatment TCZ was tolerated
Araki	2019	NMOSD	19	Intravenous TCZ at 8 mg/kg was given monthly	Maximum 6 years and 8 months	Retrospective study The number of relapses decreased in all cases Among 15 patients who received TCZ for more than twelve months: The ARR decreased markedly from 2.2 ± 1.1 to 0.3 ± 0.7 (*P*<0.001); the EDSS improved significantly from 4.5 ± 1.8 to 3.8 ± 1.4 (*P*<0.05); neuropathic pain and general fatigue also improved significantly (*P*<0.001) TCZ was well tolerated
Lotan et al.	2019	NMOSD 7 AQP4 Ab-positive 2 MOG Ab-positive 3 seronegative	12	Subcutaneous TCZ at a dose of 162 mg every 1–2 weeks	31.8 months	Retrospective study The median relapse rate was significantly reduced from 2 to 0 (*P*=0.04) The ARR decreased from a median of 2 to 0 (*P*=0.0015) One TCZ-treated patient died
Zhang et al.	2020	NMOSD 103 AQP4 Ab-positive	118	Intravenous TCZ at 8 mg/kg was given monthly Oral AZA reached the daily target dose (2–3 mg/kg)	90 weeks	Open-label, randomized phase 2 trial The median time to the first relapse was longer in the TCZ group than in the AZA group (*P*=0·0026) A total of 89% of patients treated with TCZ were relapse-free compared with 56% for treatment with AZA at 60 weeks (*P*<0.0001) Adverse events occurred in 61% of TCZ-treated patients and 83% of AZA-treated patients Two patients died but the deaths were not treatment-related
Xie et al.	2020	NMOSD	89	Intravenous TCZ at 6 mg/kg or 8 mg/kg monthly Subcutaneous TCZ at a dose of 162 mg every 1–2 weeks		Meta-analysis The ARR ratio was significantly lower in the TCZ therapy group (*P*<0.001) A significant correlation was observed between the proportion of patients with relapse-free NMOSD and TCZ therapy (OR=67.78; *P*<0.001) TCZ was well tolerated
Xue et al.	2020	NMOSD	775	485 patients were treated with monoclonal antibodies 290 were given a placebo		Meta-analysis Satralizumab and tocilizumab treatment reduced the EDSS score relative to treatment with other monoclonal antibodies (*P*=0.02) TCZ was well tolerated
Zeng et al.	2021	AQP4 Ab-positive NMOSD	50	Intravenous TCZ at 8 mg/kg was given monthly Oral AZA reached the daily target dose (2–3 mg/kg) RTX dose according to CD19+ B-cell counts	48 weeks	Retrospective study Compared with AZA, TCZ (*P*=0.012) and RTX (*P*=0.015) delayed macular ganglion cell complex thinning in the eyes of patients without a history of optic neuritis TCZ was well tolerated
Du et al.	2021	NMOSD	19	Intravenous TCZ at 8 mg/kg within 2 weeks of attack onset, and then at 4-week intervals Prednisone 10–15 mg/day for maintenance	12 months	Retrospective study Compared with steroids, patients treated with TCZ displayed significant improvements in the EDSS score, HAI, mRS, ADL, and EQ-5D-3L at 3 months (*P*<0.05) Compared with steroids, TCZ significantly lowered the risk of relapse (*P*=0.017) as well as the ARR (*P*=0.013). TCZ was well tolerated
Kharel et al.	2021	NMOSD	202	Intravenous TCZ at 6 mg/kg or 8 mg/kg monthly Subcutaneous TCZ at a dose of 162 mg every 1–2 weeks		Meta-analysis 76.95% of patients treated with tocilizumab were relapse-free at follow-up (*P*<0.001) The ARR was significantly reduced (*P*<0.001) TCZ was well tolerated
Carreón Guarnizo et al.	2022	NMOSD	5	Intravenous TCZ at 8 mg/kg every 4 weeks.	Mean duration was 2.3 ± 1 years	Retrospective study The mean ARR was reduced by 88.9% during the first year of treatment (from 1.8 ± 1.3 to 0.2 ± 0.4, *P*<0.05) TCZ was well tolerated
Novi et al.	2019	MOGAD	1	Intravenous TCZ at 8 mg/kg was given monthly	24 months	Case report No relapses occurred and spinal MRI showed a reduction in cervical and thoracic lesions at a 24-month follow-up TCZ was well tolerated
Hayward-Koennecke et al.	2019	MOGAD	1	Intravenous TCZ at 8 mg/kg was given monthly	12 months	Case report No further relapses occurred, MRI remained stable throughout, and no new lesions were detected TCZ was well tolerated
Masuccio et al.	2020	MOGAD	1	Intravenous TCZ at 8 mg/kg was given monthly	9 months	Case report After TCZ treatment, the clinical situation of the patient did not deteriorate (EDSS score: 6.5) The patient retained the ability to walk TCZ was well tolerated
Elsbernd et al.	2021	MOGAD	10	Intravenous TCZ at 8 mg/kg per month or a subcutaneous dose of 162 mg per week	28.6 months	Retrospective study None of the patients had clinical or radiographic relapses over an average treatment duration of 28.6 months TCZ was well tolerated
Ringelstein et al.	2021	14 MOGAD 36 AQP4 Ab-positive NMOSD 7 seronegative NMOSD	57	Intravenous dose of 8.0 mg/kg (median; range 6–12 mg/kg) every 31.6 days	23.8 months	Case-series 60% of all patients were relapse-free (79% for those with MOGAD) The median ARR decreased from 1.75 to 0 (*P*=0.0011) and inflammatory activity on MRI decreased in patients with MOGAD who received TCZ (*P*=0.04) 11 of the patients with MOGAD who received tocilizumab for more than 12 months had reduced ARR, and 73% of these were relapse-free TCZ was well tolerated
Sato et al.	2014	MS	1	Intravenous TCZ at 8 mg/kg was given monthly	5 years	Case report Complete remission was achieved at the second administration of TCZ. No recurrence of MS for more than 5 years TCZ was well tolerated
Hoshino et al.	2020	MS	1	Intravenous TCZ at 8 mg/kg was given monthly	2 years	Case report One year and 6 months after TCZ therapy, the oral PSL dose was tapered from 30 to 10 mg/day and the EDSS score improved from 8.5 to 5.0 Five years from the disease onset, brain MRI showed regression of the known lesions TCZ was well tolerated
Yamamura et al.	2019	NMOSD	83	Subcutaneous satralizumab 120 mg or matching placebo at weeks 0, 2, and 4 and then once every 4 weeks	216 weeks	Randomized, double-blind, placebo-controlled phase III study Relapse occurred in 20% of patients receiving satralizumab and in 43% of patients receiving a placebo Among 55 AQP4-IgG-seropositive patients, the relapse rates were 11% in the satralizumab group and 43% in the placebo group No significant differences in neuropathic pain or fatigue were observed between the satralizumab and placebo groups Satralizumab was well tolerated
Traboulsee et al.	2020	NMOSD	95	Subcutaneous satralizumab 120 mg at weeks 0, 2, and 4 and then once every 4 weeks	216 weeks	Randomized, double-blind, placebo-controlled phase III study Relapses occurred in 30% of patients receiving satralizumab and in 50% of patients receiving a placebo For the AQP4-IgG-seropositive subgroup, relapse occurred in 22% of patients receiving satralizumab *versus* 57% for the placebo No significant differences in neuropathic pain or fatigue were observed between the satralizumab and placebo groups Satralizumab was well tolerated
Uzawa et al.	2022	NMOSD	1	Subcutaneous satralizumab 120 mg at weeks 0, 2, and 4 and then once every 4 weeks	12 months	Case report ARR was reduced from 2.3 to 0 times/year Painful tonic seizures disappeared after 6 months Satralizumab was well tolerated

CNS IDDs, central nervous system inflammatory demyelinating diseases; TCZ, tocilizumab; AQP4, Aquaporin 4; NMOSD, neuromyelitis spectrum disorders; MOGAD, myelin oligodendrocyte glycoprotein antibody-associated disease; MS, multiple sclerosis; ARR, annualized relapse rate; EDSS, Expanded Disability Status Scale score; HAI, Hauser ambulation index; mRS, modified Rankin score; ADL, activities of daily living; EQ-5D-3L, EuroQol five-dimension three-level questionnaire; AZA, azathioprine; PSL, prednisolone.

The pathogenic effect of IL-6 signaling in MS may be exerted through the induction of IL-17-producing T cells. The effect of lL-6 inhibition in MS treatment is unclear. Evidence from mouse models of MS (experimental autoimmune encephalomyelitis) indicates that Th17 cells play an important role in MS progression (74, 75). In the mouse, IL-6 mainly induce the differentiation of naive CD4-positive T cells into Th17 cells, whereas anti-IL-6 therapy effectively suppresses the onset of experimental autoimmune encephalomyelitis *via* the inhibition of the development of autoantigen-specific Th17 cells. In humans, the data are limited to a few studies. One patient with RA and MS received tocilizumab for more than 5 years without exacerbation of MS (62), whereas another RA patient developed MS during tocilizumab treatment (76). Although this result indicates that IL-6 inhibition might trigger inflammatory demyelination in the CNS, elevated levels of the soluble IL-6 receptor, an indirect marker for reduced IL-6 signaling, were found to be significantly correlated with a reduced risk of MS. This strongly suggests that IL-6 receptor inhibitor therapy may be suitable for use in MS treatment (77). Additionally, there are two recent case reports of patients with MS who have been successfully treated with tocilizumab (62, 63) (**Figure 1**, **Table 1**). Accordingly, the role of IL-6 in MS pathogenesis and the efficacy of IL-6 receptor inhibitors in MS treatment merit further evaluation.

Biopsy and autopsy data demonstrate complement and immunoglobulin deposition in demyelinating lesions of patients with MOGAD, indicative of a significant humoral immune component similar to that seen in AQP4 antibody-positive NMOSD (5, 15, 78). Although NMOSD and MOGAD have different underlying pathogenic mechanisms, they are both characterized by elevated levels of IL-6 in serum and CSF, especially during relapses (57, 79–83). The similarity in CSF cytokine profiles provides promising therapeutic options for NMOSD and MOGAD using IL-6 inhibitors. To date, relatively few reports have assessed the efficacy of tocilizumab treatment on patients with MOGAD (48, 53–57, 82, 83), (**Figure 1**, **Table 1**), and further studies are needed to confirm the efficacy and safety of anti-IL-6 receptor therapy in the treatment of this disease.

### Intravenous or subcutaneous tocilizumab

Lotan et al. (48) reports 12 NMOSD patients who received at least 6 months of subcutaneous tocilizumab. The ARR decreased from a median of 2 (5.75–1.29) before subcutaneous tocilizumab to 0 (1.0–0) (*P*=.0015) after treatment. The efficacy of subcutaneous tocilizumab in NMOSD was similar to that of the intravenous formulation.

One case series reports seven patients (four with NMOSD and three with MOGAD), all of whom first received tocilizumab by intravenous injection; subsequently, three patients switched to the subcutaneous form of administration. None of these patients relapsed during tocilizumab treatment (59). Another case series describes 10 patients with relapsing MOGAD treated with tocilizumab, including six by intravenous injection, two by subcutaneous injection, and two who changed from intravenous to subcutaneous injection. All the patients were relapse-free during 28.6 months of follow-up (60), indicating that intravenous and subcutaneous tocilizumab have similar clinical efficacy. Similar results were reported by Lotan et al. (48).

The use of subcutaneous injection should be encouraged given its advantage of at-home administration. Prospective studies of subcutaneous tocilizumab treatment for CNS IDDs are warranted.

### The usage of IL-6 receptor antagonists in pregnancy

Hoeltzenbein et al. undertook an analysis of a global safety database containing data for 399 women exposed to tocilizumab shortly before or during pregnancy, 288 of whom reported pregnancy outcomes. Of these 288 pregnancies, 60.6% resulted in live births, 21.7% in spontaneous abortions, 17.2% in elective terminations, and one in stillbirth. The rate of deformity was 4.5%. Although the rate of preterm birth increased (31.2%) compared with the general population, no substantial increase in the risk for deformity was observed (84). In a retrospective analysis from Japan, the authors analyzed pregnancy outcomes in patients with rheumatic disease exposed to tocilizumab. No increased risk of spontaneous abortion or congenital malformation was found in 61 pregnancies (85).

Data regarding the safety of tocilizumab during pregnancy and breastfeeding among patients with RA are limited, and these patients are advised to stop tocilizumab treatment 3 months before conception (86). In NMOSD, experts advise that tocilizumab can be used by pregnant women with severe disease, whereas breastfeeding may be considered, but only under close monitoring (87). The effects of satralizumab treatment on pregnancy outcomes are unknown.

### Analysis of the cost effectiveness of IL-6 receptor antagonists

A cost-utility analysis of tocilizumab in the treatment of patients with RA in Japan showed that quality-adjusted life years (QALYs) and lifetime costs of tocilizumab were approximately 1.3- and 1.5-fold higher, respectively, compared with those for methotrexate. The incremental cost per QALY for tocilizumab treatment was reported to be USD 49,359, which was below the assumed cost-effectiveness threshold of USD 50,000 per QALY (88). These findings indicated that tocilizumab may be cost-effective in the treatment of RA.

A cost-effectiveness analysis of tocilizumab in patients with RA from the United Kingdom, Greece, and Italy showed similar results; namely, that tocilizumab, used either as a first-line biologic monotherapy or an addition to the standard treatment strategy, can be a cost-effective option for the treatment of patients with RA who have not adequately responded to conventional drugs (89–91).

No economic evaluation of tocilizumab and satralizumab therapy in the treatment of CNS IDDs has been undertaken to date. An assessment of the cost-effectiveness of IL-6 receptor inhibitors is warranted in the near future.

Finally, the cost of satralizumab in the United States is USD 219,231 for the first year, subsequently decreasing to USD 190,000 per year (92). The high costs of these new drugs limit their usage in low-income countries, and finding ways of providing these drugs at more reasonable prices constitutes a major challenge for the governments of these countries.

## Author contributions

LJ conceived and wrote the manuscript, SG read and approved the submitted version.

## Funding

This work was supported by the National Natural Science Foundation of China (Grant no. 82072079).

## Conflict of interest

The authors declare that the research was conducted in the absence of any commercial or financial relationships that could be construed as a potential conflict of interest.

## Publisher’s note

All claims expressed in this article are solely those of the authors and do not necessarily represent those of their affiliated organizations, or those of the publisher, the editors and the reviewers. Any product that may be evaluated in this article, or claim that may be made by its manufacturer, is not guaranteed or endorsed by the publisher.
